# Determinants of exclusive breastfeeding practices among mothers in Ethiopia

**DOI:** 10.1371/journal.pone.0281576

**Published:** 2023-02-09

**Authors:** Mitiku Wale Muluneh

**Affiliations:** Department of Statistics, Debre Tabor University, Debre Tabor, Ethiopia; Aga Khan University pakistan, PAKISTAN

## Abstract

**Background:**

Exclusive breastfeeding (EBF) is the most cost-effective intervention to reduce infant morbidity and mortality worldwide. It is crucial since human milk contains nutrients, living cells, and defensive factors which enable infants to have better immunity, physical and mental development. Therefore, this study aimed at identifying the predictors of exclusive breastfeeding practices among mothers in Ethiopia using Ethiopian Demographic and Health Survey (EDHS) 2016 data.

**Methods:**

EDHS 2016 data were used for the analysis. A total of 1,066 mothers were included in the analysis. The binary logistic regression model was used to identify the determinants of EBF practice among mothers. The result presented using adjusted odd ratio (AOR) with a 95% confidence interval.

**Results:**

The prevalence of EBF was 58% of infants under age 6 months. Mothers age 25–34 (AOR = 1.74; 95% CI 1.31–2.32), child age 4–5 months (AOR = 0.74; 95% CI 0.66–0.84), married marital status (AOR = 1.26; 95% CI 1.06–1.50), mothers attained secondary education or higher (AOR = 2.00; 95% CI 1.54–2.58), husband attained secondary education or higher (AOR = 1.70; 1.39–2.13), richer wealth index (AOR = 0.35; 95% CI 0.18–0.69), accessed to the media (AOR = 1.77; 95% CI 1.38–2.27), number of living children 3–4 (AOR = 0.49; 95% CI 0.25–0.95), health facility (AOR = 1.87; 95% CI 1.09–3.20), rural residence (AOR = 0.66; 95% CI 0.49–0.89) and mothers living in Afar (AOR = 100.2; 95% CI 15.68–640.61), Somali (AOR = 52.65; 95% CI 8.48–326.77), SNNPR (AOR = 6.94; 95% CI 1.05–45.79), Harari (AOR = 61.94; 95% CI 9.75–393.44), Addis Ababa (AOR = 13.07; 95% CI 2.06–82.99), and Dire Dawa (AOR = 28.91; 95% CI 4.38–190.86) were associated with EBF practice.

**Conclusions:**

The practice of exclusive breastfeeding remains low in Ethiopia. Therefore, the stakeholders should be taken into consideration those determinant factors identified in this study in policies and programmes to increase EBF practice among mothers. Moreover, designing and implementing specific strategies to enhance the rate of exclusive breastfeeding practices through community-based education is recommended.

## Background

Exclusive breastfeeding (EBF) is the practice of feeding infants only breast milk for the first six months [1,2]. It is the most cost-effective intervention to reduce infant morbidity and mortality worldwide [3,4]. Breast milk is a safe and nutritive diet for the healthy growth and development of infants. Exclusive breastfeeding (EBF) for the first six months and continued breastfeeding for two years is recognized by the World Health Organization (WHO), with the introduction of supplemental food at six months of age [5]. However, only 40% of infants under six months of age are exclusively breastfed globally [6].

Breastfeeding is an important method for improving public health as it has numerous documented health advantages for babies and mothers [7]. For babies, it offers irreplaceable nutrients for growth and development, decreases the risk of becoming overweight or obese, and protects against certain non-communicable diseases later in life, helps with brain and nervous system development, as well as having higher intelligence quotients (IQs). It can also protect the child from respiratory infections, diarrheal sickness, and other potentially life-threatening illnesses. Allergies, colds, diaper rashes, and ear and stomach disorders are less common in breastfed babies. For the mother, it helps the uterus contract to return to its normal size, and it creates strong bonding between a mother and a baby. Breastfeeding for longer periods of time benefits mothers’ health and well-being by lowering the risk of ovarian and breast cancer and spacing pregnancies. Breastfeeding is convenient, costs less than formula and a secure way of feeding and is safe for the environment [7–9]. Despite the world’s commitment to reduce child mortality by two-thirds by 2015 from 1990 levels, under-five mortality has decreased by half in the last two decades, from 12.7 million to 6.3 million in 2013. Around 180 countries have committed to stepping up efforts to decrease maternal, neonatal, and child deaths as part of the Sustainable Development Goals (SDGs) in order to retain the momentum of recent successes [10]. EBF practices can reduce infant mortality rates by 13% in low-income nations like Bangladesh and Pakistan [11], in addition to infant and maternal health benefits [12–14].

Breastmilk confers short- and long-term benefits to both child and mother [15], tends to increase the mother-child relationship, develops immunity, and saves money on artificial milk [16–19]. EBF is crucial since human milk contains nutrients, living cells, and defensive factors which enable infants to have better immunity, physical and mental development. However, more than 85% of mothers worldwide did not follow the WHO recommendation, and only 35% of infants younger than four months were exclusively breastfed. Evidence shows that the majority of mothers started to EBF their infants at birth and the rate declined greatly for about two or more months [20]. Trend data analysis on the prevalence of exclusive breastfeeding among infants less than 6 months of age in developing countries indicated that only 39% of the infants were exclusively breastfed in 2010. The estimated prevalence of EBF in Africa and Asia is 35 and 41% respectively [21]. Sub-Saharan Africa (SSA) continues to shoulder the greatest burden where the under-five deaths are 15 times higher than deaths in an average high-income country [10].

In Ethiopia, according to the results of the Ethiopian Demographic and Health Survey (EDHS), 2016 report almost all children (97%) are breastfed at some point. However, only 58% of infants under age 6 months are exclusively breastfed in 2016 [22]. Several factors can affect the pattern of EBF practice that can be further classified as an infant, maternal and household characteristics, and health service-related factors [23–26]. In Ethiopia, infant characteristics including age and prolateral feeds; maternal characteristics such as age, education, occupation, and marital status; and health facility characteristics including place of delivery, antenatal and postnatal care, and counseling were previously reported to influence EBF [10,20,27–29]. Strategies to promote exclusive breastfeeding are desired at the national, health facility, and community levels [30]. Exclusive breastfeeding is important for child health and growth, but its practice is low in many developing countries [31]. Therefore, this study aimed at identifying the predictors of exclusive breastfeeding practices among mothers in Ethiopia using EDHS 2016 data.

## Materials and methods

### Study setting and design

This study was carried out in Ethiopia using EDHS 2016 data to assess the factors associated with EBF practice among mothers in Ethiopia. Ethiopia is located on the Horn of Africa (3°- 14°N and 33°-48°E). The country covers 1.1 million square kilometers and has a high central plateau that varies from 4550 m above sea level to the far depression to 110 m below sea level. Ethiopia is divided into nine administrative regions and two city administrations. The regions are organized into 68 zones, each of which has 817 administrative districts. EDHS 2016 was a recent community based cross-sectional survey conducted across the country [22,32].

### Data source and population

The EDHS is a national survey intended to provide demographic and health indicators. A two-stage stratified cluster sampling procedure was used for the survey. In the first stage, 645 enumeration areas were selected using systematic sampling with probability proportionate to size (202 in urban areas). In the second stage, a fixed number of 28 households per cluster were randomly selected from the household listing [22,32]. The source of the population for this study was all women aged 15 to 49 from the individual woman questionnaire. The analysis focused on women who had less than six (6 months) age children before the collection and who consented to the survey during the collection period. This study included all mothers of children who were less than six months old at the time of data collection, whether or not they used exclusive breastfeeding, were present during the investigators’ visit, and answered the breastfeeding question. The total number of interviewed mothers were 15683. However, this study considers mothers who have a child and a child age less than six months. In addition, mothers who have missing values exclude from the study. Therefore, a total sample of 1,066 women was included in this study.

### Variables of study

#### Dependent variable

The dependent variable for this study was exclusive breastfeeding of infants less than 6 months of age. All mothers who answered that they had not given their child any food other than breast milk 24 h preceding the survey was coded 1 and the modality which corresponds to the ingestion of foods other than breast milk for the child was coded 0.

#### Independent variables

Many independent variables were assessed as potential factors that influence exclusive breastfeeding. Variables are age of the child in months, sex of child, mother’s age, mother educational level, husband educational level, marital status, number of living children, religion, wealth index, working status, antenatal care visits, place of delivery, access to mass media, place of residence and region. These variables were considered because of their statistically significant relationships with EBF in previous studies [10,27,33].

#### Methods of data analysis

Data were extracted using SPSS version 21 software and then exported to the R version 4.1.2 statistical software for further analysis. Descriptive statistics including frequencies and percentages were performed to describe study participants. A stepwise logistic regression model was used to identify factors associated with EBF. All variables with p values <0.05 have been considered statistically significant.

Data were extracted using the SPSS version 21 software and then exported to R version 4.0.3 statistical software for further analysis. Descriptive statistics including frequencies and percentages were computed to describe the study participants. A stepwise binary logistic regression model was used to identify factors associated with desire for more children. The results were presented as adjusted odds ratio (AOR) together with their corresponding 95% confidence intervals signifying the level of precision. Multicollinearity was tested using the variance-inflation factor (VIF) test, suggesting that there was no multicollinearity since all variables had VIF< 5. To determine the goodness of fit is through the Homer-Lemeshow statistics, which is computed on data after the observations have been segmented into groups based on having similar predicted probabilities. Therefore, in this study, the Homer-Lemeshow test was greater than 0.05 (P-value > 0.05), indicating that the model was a good fit [34].

### Ethical consideration

The study was used secondary data analysis of publicly available survey data from the DHS program, ethical approval, and participant consent was not necessary for this study. Permission to access the data set was obtained from the Measure DHS International Program. The data was only used for purpose of this study and not shared with a third party. All data used in this study were anonymous publicly available and aggregated secondary data with not having any personal identity. The data was fully available on the full DHS website (www.measuredhs.com).

## Results

### The characteristics of the respondents

A total of 1066 mothers were included in the study. Only 58% of infants under age 6 months are exclusively breastfeed. Most mothers (48.8%) were in the 25–34 age group. 50.8% of children were females and 49.2% of children were males. Most of the study respondents were from rural residences (80.6%). About 95.6% of mothers were married. About 58.3% of mothers did not attend primary school and only 13.7% of the mothers attended secondary and above level education. About 45.6% of the husbands did not attend primary school and only 22.8% of the husbands attended secondary and higher-level education. About 37.9% of mothers had the poorest wealth index, 16.0% of mothers had a poorer wealth index, 11.7% of mothers had a middle wealth index, 13.7% of mothers were richer, and others had the richest wealth index 20.8%. Most of the respondents were housewives/not working (80.6%) and 19.4% of the respondents were employed/workers. Above half of the mothers (51.0%) were Muslim religious followers. The largest and the least proportion 15.9% and 4.5% of respondents were from the Oromia regional state and the Dire Dawa city administration, respectively. The majority (65.0%) of the respondents had no access to the media. About 47.5% of the respondents had 3–4 living children. Of the total of the respondents, 56.9% of the respondents gave child at home place of delivery, and 68.1% of the respondents had ANC visits (Table 1).

**Table 1:** Result of mother’s characteristics of exclusive breastfeeding in Ethiopia, EDHS 2016.

Variable	Category	Frequency	Percent
Mother age in years	15–24 25–34 35–49	365 520 181	34.2 48.8 17.0
Child age in months	0–1 2–3 4–5	343 347 376	32.2 32.6 35.3
Sex of child	Male Female	525 541	49.2 50.8
Residence	Urban Rural	207 859	19.4 80.6
Marital status	Unmarried Married	47 1019	4.4 95.6
Mothers’ education	No education Primary education Secondary and above	622 298 146	58.3 28.0 13.7
Husband education	No education Primary education Secondary and above	486 337 243	45.6 31.6 22.8
Wealth index	Poorest poorer Middle Richer Richest	404 171 125 144 222	37.9 16.0 11.7 13.5 20.8
Mothers’ occupation	Not working/housewife Workers/employed	859 207	80.6 19.4
Religion	Orthodox Muslim Protestant Others	319 544 179 24	29.9 51.0 16.8 2.3
Region	Tigray Afar Amhara Oromia Somali Benishangul SNNPR Gambela Harari Addis Ababa Dire Dawa	115 100 100 170 149 88 122 64 60 50 48	10.8 9.4 9.4 15.9 14.0 8.3 11.4 6.0 5.6 4.7 4.5
Access to media	No Yes	693 373	65.0 35.0
Number of living children	1–2 3–4 ≥5	237 506 323	22.2 47.5 30.3
Place of delivery	Home Health facility	607 459	56.9 43.1
ANC visits	No Yes	340 726	31.9 68.1

### Determinants of exclusive breastfeeding practices in Ethiopia

The odds of EBF practices to their child among mothers age 25–34 were 1.74 (AOR = 1.74; 95% CI 1.31–2.32) times higher compared to mothers age group 15–24 years. The odds of EBF to their child among mothers whose child age was 4–5 months were 0.74 (AOR = 0.74; 95% CI 0.66–0.84) times lower compared to mothers whose child age was 0–1 months. Mothers who are currently married were 1.26 (AOR = 1.26; 95% CI 1.06–1.50) times more likely to breastfeed their child exclusively compared to mothers who are not currently married. The odds of EBF to their child among mothers who had primary and secondary education or higher were 1.41 (AOR = 1.41; 95% CI 1.20–1.66) and 2.00 (AOR = 2.00; 95% CI 1.54–2.58) times higher compared to mothers who did not have formal education, respectively. Mothers whose husbands had primary and secondary education or higher were 1.50 (AOR = 1.56; 95% CI 1.28–1.75) and 1.70 (AOR = 1.70; 1.39–2.13) times more likely to breastfeed their child exclusively compared to mothers whose husband did not have formal education, respectively. Mothers whose wealth index was poorer, middle, and richer were 0.24 (AOR = 0.24; 95% CI 0.09–0.61), 0.12 (AOR = 0.12; 95% CI 0.06–0.25) and 0.35 (AOR = 0.35; 95% CI 0.18–0.69) times less likely to breastfeed their child exclusively compared to mothers whose wealth index was poorest, respectively. Mothers who had accessed the media were 1.77 (AOR = 1.77; 95% CI 1.38–2.27) times more likely to breastfeed their child exclusively compared to mothers who did not have access to the media. Mothers who had 3–4 children were 0.49 (AOR = 0.49; 95% CI 0.25–0.95) times less likely to breastfeed their child exclusively compared to mothers who have 1–2 children. Mothers who gave birth at a health facility practiced exclusive breastfeeding were 1.87 (AOR = 1.87; 95% CI 1.09–3.20) times higher compared to mothers who gave birth at home. The odds of EBF to their child among mothers who lived in the rural areas were 0.66 (AOR = 0.66; 95% CI 0.49–0.89) times lower compared to mothers who lived in the urban areas. The odds of EBF to their child among mothers living in Afar, Somali, SNNPR, Harari, Addis Ababa, and Dire Dawa regions were 100.2 times (AOR = 100.2; 95% CI 15.68–640.61), 52.65 times (AOR = 52.65; 95% CI 8.48–326.77), 6.94 times (AOR = 6.94; 95% CI 1.05–45.79), 61.94 times (AOR = 61.94; 95% CI 9.75–393.44), 13.07 times (AOR = 13.07; 95% CI 2.06–82.99), and 28.91 times (AOR = 28.91; 95% CI 4.38–190.86) higher compared to mothers living in Tigray region (Table 2).

**Table 2:** Factors associated with EBF practice among mothers in Ethiopia, EDHS 2016.

Variables	Category	AOR (95% CI)
Mother age in years	15–24 25–34 35–49	1 1.74(1.31–2.32) * 3.71(2.80–4.92) *
Child age in months	0–1 2–3 4–5	1 0.71(0.40–1.24) 0.74(0.66–0.84) *
Sex of child	Male Female	1 0.69(0.45–1.07)
Marital status	Unmarried Married	1 1.26(1.06–1.50) *
Mother education	No education Primary education Secondary and above	1 1.41(1.20–1.66) * 2.00(1.54–2.58) *
Husband education	No education Primary education Secondary and above	1 1.50(1.28–1.75) * 1.70(1.39–2.13) *
Wealth index	Poorest poorer Middle Richer Richest	1 0.24(0.09–0.61) * 0.12(0.06–0.25) * 0.35(0.18–0.69) * 0.32(0.08–1.23)
Mother’s occupation	Not working/Housewife Workers /employed	1 0.52(0.31–0.87) *
Religion	Orthodox Muslim Protestant Others	1 0.44(0.17–1.12) 0.31(0.09–1.13) 0.87(0.45–1.68)
Access to mass media	No Yes	1 1.77(1.38–2.27) *
Number of living children	1–2 3–4 ≥5	1 0.49(0.25–0.95) * 0.45(0.19–1.10)
Place of delivery	Home Health facility	1 1.87(1.09–3.20) *
ANC visits	No Yes	1 2.00(1.17–3.41)
Residence	Urban Rural	1 0.66(0.49–0.89) *
Region	Tigray Afar Amhara Oromia Somali Benishangul SNNPR Gambela Harari Addis Ababa Dire Dawa	1 100.2(15.68–640.61) * 3.40(0.61–18.78) 2.21(0.32–15.42) 52.65(8.48–326.77) * 1.00(0.08–12.63) 6.94(1.05–45.79) * 2.05(0.19–22.34) 61.94(9.75–393.44) * 13.07(2.06–82.99) * 28.91(4.38–190.86) *

1 shows the reference category and

* shows the reference p-value < 0.05.

## Discussion

The purpose of this study was to determine the individual and contextual factors of the practice of exclusive breastfeeding in Ethiopia. The prevalence of EBF in this study was 58%. This study showed that the practice of exclusive breastfeeding was associated with mothers’ age. The result showed that older mothers practiced more EBF compared to younger mothers of age.

This finding was consistent with another study done in Ethiopia [20]. The probable reason could be that as the maternal age increases, infant management experiences will be increased. Younger mothers believed that practicing EBF for a longer time would impact their breast size and beauty. Infant age was significantly associated with EBF practices. The practice of EBF decreased significantly the age of infants close to the first six months. This study was consistent with the previous studies done in Ethiopia, Malawi, and Brazil [35–37]. This could be explained by the traditional postpartum family care given in the first few months when mothers remain at home, which might encourage exclusive breastfeeding [29]. Complementary feeding might be introduced to infants with the assumption that breast milk alone will not satisfy the needs of infants as they approach six months of age [38].

In this study, the marital status of the mother was identified as the associated factor with practice EBF. The result showed that married mothers were more likely to practice EBF compared to unmarried mothers. This result was consistent with the other studies conducted in Tanzania, and Canada [39–41]. The probable reason could be married mothers get support to practice EBF from their partners and other family members. Mothers and husbands with no education were less likely to exclusively breastfeed their infants compared to educated mothers and husbands. This study is similar to the other studies done in Ethiopia, and Brazil [20,42,43]. This could be because no education mothers lack scientific knowledge and have trouble comprehending EBF’s written messaging and antenatal care advice. Hence, they will introduce supplementary feeding to their infants early by assuming it is good compared with the educated mothers [44].

In the current study, the household wealth index was significantly associated with EBF. This finding is similar to other studies done in Ghana, India, and Maharashtra [45–47]. This could be attributed to a greater understanding of breastfeeding-related information and improved skills in negotiating flexible work hours, including the ability to stay at home and breastfeed exclusively [48]. Similarly, the low use of EBF among the poorest wealth index could be due to a lack of awareness of the technique, as well as a stressful living environment. In addition, mothers who are housewives were more likely to practice EBF than employed mothers. This result is similar to other studies done in Ethiopia, and Malaysia [49–51]. The possible explanation may be the early return of employed mothers to the office, lack of support from the office and short maternity leave (only four months paid leave in the Ethiopian case) could also discourage employed mothers. Employed mothers may be relatively overloaded with their office and home activities and may have limited contact time with infants. Mothers who have accessed mass media infant feeding counselling had more likely to practice EBF for the recommended duration compared to those mothers who didn’t have access to mass media. This study was in line with another study done in Ethiopia [52]. The probable explanation may be mothers who have accessed information about EBF practice have a better knowledge of the advantage of exclusive breastfeeding practice to child mental and physical health [53].

Furthermore, the number of living children was positively associated with EBF practice. This indicates that mothers who have 3–4 children were less likely to practice EBF than mothers who have 1–2 children. Place of delivery was significantly associated with EBF practice. That is mothers who delivered at health facilities practiced EBF more likely than mothers who delivered at home. This study was consistent with the other studies done in Ethiopia, and Uganda [10,20,24]. This could be explained by the fact that mothers who give birth in health facilities have better access to breastfeeding resources, like as counselling on the benefits of breastfeeding, proper positioning, and bonding [54].

Finally, the study found that mothers living in rural areas had less likely exclusive breastfeeding practice compared to those mothers living in urban areas. This study was consistent with another study done in Ethiopia [6]. The probable explanation is the rural mothers were producing little milk owing to lack of food and starvation, less access to information, health facility, and lack EBF education for their children [10]. This study revealed that the region was significantly associated with the practice of exclusive breastfeeding to their child. Mothers living in Afar, Somali, SNNPR, Harari, Addis Ababa, and Dire Dawa administrative regions were more likely to exclusively breastfeed compared to those mothers living in the Tigray region. The variation in this study could be regional differences in some background characteristics such as culture, religion, living conditions, values of the community, availability, and accessibility of maternal and child health services [55].

## Conclusion

The practice of exclusive breastfeeding remains low in Ethiopia. The age of mothers, age of the child, marital status of mothers, mothers’ educational status, husband educational status, wealth index, mother occupation, access to mass media, number of living children, and place of delivery, place of residence and region were associated factors with EBF. Therefore, the stakeholders should be taken into consideration those determinant factors identified in this study in policies and programmes to increase EBF practice among mothers. Moreover, designing and implementing specific strategies to enhance the rate of exclusive breastfeeding practices through community-based EBF education is recommended.

## Limitation of the study

This study used secondary data. The data have missing value in the variable and some variables are not found in the dataset which are some important variables missing from the analysis. Therefore, missingness in the data and variables were the main limitation of this study.

## Abbreviations

ANC: Antenatal Care
AOR: Adjusted Odds Ratio
CI: Confidence Interval
EBF: Exclusive Breastfeeding
EDHS: Ethiopian Demographic and Health Survey
IRB: Institutional Review Board
SNNPR: Southern Nations, Nationalities, and Peoples’ Region
